# BPD-MA-mediated photosensitization in vitro and in vivo: cellular adhesion and β_1_ integrin expression in ovarian cancer cells

**DOI:** 10.1038/sj.bjc.6690448

**Published:** 1999-06

**Authors:** J M Runnels, N Chen, B Ortel, D Kato, T Hasan

**Affiliations:** Department of Dermatology, Wellman Laboratories of Photomedicine, Massachusetts General Hospital – WEL 224, Boston, MA, 02114, USA; Vincent Gynecologic Oncology Service, Massachusetts General Hospital – VBK-1, Boston, MA, 02114, USA

**Keywords:** BPD-MA, photosensitization, cellular adhesion, ECM proteins, OVCAR 3, ovarian cancer

## Abstract

Benzoporphyrin derivative monoacid (BPD-MA) photosensitization was examined for its effects on cellular adhesion of a human ovarian cancer cell line, OVCAR 3, to extracellular matrix (ECM) components. Mild BPD-MA photosensitization (~ 85% cell survival) of OVCAR 3 transiently decreased adhesion to collagen IV, fibronectin, laminin and vitronectin to a greater extent than could be attributed to cell death. The loss in adhesiveness was accompanied by a loss of β_1_ integrin-containing focal adhesion plaques (FAPs), although β_1_ subunits were still recognized by monoclonal antibody directed against human β_1_ subunits. In vivo BPD-MA photosensitization decreased OVCAR 3 adhesiveness as well. Photosensitized adhesion was reduced in the presence of sodium azide and enhanced in deuterium oxide, suggesting mediation by singlet oxygen. Co-localization studies of BPD-MA and Rhodamine 123 showed that the photosensitizer was largely mitochondrial, but also exhibited extramitochondrial, intracellullar, diffuse cytosolic fluorescence. Taken together, these data show that intracellular damage mediated by BPD-PDT remote from the FAP site can affect cellular–ECM interactions and result in loss of FAP formation. This may have an impact on long-term effects of photodynamic therapy. The topic merits further investigation. © 1999 Cancer Research Campaign


*British Journal of Cancer* (1999) **80**(7), 946–953
© 1999 Cancer Research Campaign
Article no. bjoc.1998.0448

### BPD-MA-mediated photosensitization in vitro and in vivo: cellular adhesion and $\beta_1$ integrin expression in ovarian cancer cells

**JM Runnels[1], N Chen[1], B Ortel[1], D Kato[2] and T Hasan[1]**

[1]Wellman Laboratories of Photomedicine, Department of Dermatology, Massachusetts General Hospital – WEL 224, Boston, MA 02114, USA;
[2]Vincent Gynecologic Oncology Service, Massachusetts General Hospital – VBK-1, Boston, MA 02114, USA

**Summary** Benzoporphyrin derivative monoacid (BPD-MA) photosensitization was examined for its effects on cellular adhesion of a human ovarian cancer cell line, OVCAR 3, to extracellular matrix (ECM) components. Mild BPD-MA photosensitization (~ 85% cell survival) of OVCAR 3 transiently decreased adhesion to collagen IV, fibronectin, laminin and vitronectin to a greater extent than could be attributed to cell death. The loss in adhesiveness was accompanied by a loss of $\beta_1$ integrin-containing focal adhesion plaques (FAPs), although $\beta_1$ subunits were still recognized by monoclonal antibody directed against human $\beta_1$ subunits. In vivo BPD-MA photosensitization decreased OVCAR 3 adhesiveness as well. Photosensitized adhesion was reduced in the presence of sodium azide and enhanced in deuterium oxide, suggesting mediation by singlet oxygen. Co-localization studies of BPD-MA and Rhodamine 123 showed that the photosensitizer was largely mitochondrial, but also exhibited extramitochondrial, intracellular, diffuse cytosolic fluorescence. Taken together, these data show that intracellular damage mediated by BPD-PDT remote from the FAP site can affect cellular–ECM interactions and result in loss of FAP formation. This may have an impact on long-term effects of photodynamic therapy. The topic merits further investigation.

**Keywords:** BPD-MA; photosensitization; cellular adhesion; ECM proteins; OVCAR 3; ovarian cancer

Photodynamic therapy (PDT) has recently been approved by various regulatory agencies for the treatment of certain cancers (Hasan and Parrish, 1996; Dougherty et al, 1998). Investigations of cellular molecular responses to PDT reveal release of prostaglandin $E_2$ ($PGE_2$) (Henderson and Donovan, 1989), histamine release from mast cells (Glover et al, 1989), increased transcription and translation of some oxidative stress genes (Gomer et al, 1989) including haem oxygenase (Gomer et al, 1991), and the heat shock protein, Hsp70 (Gomer et al, 1988). More long-term effects, such as resistance to photosensitization (Singh et al, 1991; DiProspero et al, 1997) and mutation (Evans et al, 1989) could be the result of PDT-induced DNA single-strand breaks, sister chromatid exchanges or other chromosomal aberrations (Gomer et al, 1983).

PDT often leaves a significant number of surviving tumour cells which have been exposed to both light and photosensitizer but not enough of either to be destroyed. Our interest was an investigation of such cells, because of their potential impact on long-term effects of PDT. For example, a reduction in metastases has been reported in vivo after PDT compared to after surgery (Gomer et al, 1987), but no studies directly investigating the molecular basis of this observation have been reported. However, recent studies point to the intertwined nature of angiogenesis, tumorigenesis and extracellular matrix (ECM) metabolism (Ingber and Folkman, 1988; Brem et al, 1993). The complex nature of the mechanisms in cell and tissue damage in response to PDT could profoundly influence the metastatic process. Along these lines it was shown that PDT causes cellular membrane damage (Malik and Djaldetti, 1980; Evenson et al, 1984; Moan and Vistnes, 1986), and tumour cells treated with Photofrin® (PF®)-based PDT release compounds such as prostanoids including prostaglandin $E_2$ ($PGE_2$), prostacyclin, thromboxane (TX) and von Willebrand factor (vWf) (Henderson and Donovan, 1989; Fingar et al, 1990; Foster et al, 1991). $PGE_2$ has been shown to directly influence the in vivo dissemination and in vitro migration of Lewis lung carcinoma cells (Young et al, 1987), and differences exist in the amount of synthesis of $PGE_2$ and prostacyclin by normal, tumour and metastatic cells (Young et al, 1987). In addition, inhibition of the cyclooxygenase pathway renders tumour cells non-invasive and non-metastatic (Reich et al, 1989). TX and vWf are both involved in platelet aggregation and adhesion, which have been shown to play a role in metastasis in vivo (Karpatkin et al, 1988). Most recently, Foultier et al (1994) found that PDT using hematoporphyrin derivative (HPD-PDT) decreased the adhesiveness of colonic cancer cells to endothelial cell monolayers.

The present study focuses on one cellular response to PDT relevant to the above discussion, and that is the effect on cellular adhesion to ECM proteins. A group of cell surface proteins that contribute to cell adhesion, differentiation and malignant transformation processes are the integrins. These are heterodimeric cell surface receptors composed of α and β subunits. Generally, both subunits have long extracellular domains and small cytoplasmic domains. Integrins serve to link the external and internal cellular environments by functioning in transmembrane signalling (Schwartz, 1992). Integrin up- and down-regulation has been associated with malignant transformation (Plantefaber and Hynes, 1989; Schreiner et al, 1989; Albeda et al, 1990; Dedhar and Saulnier, 1990; Giancotti and Ruoslahti 1990; Schreiner et al, 1991) and metastasis (Plantefaber and Hynes, 1989; Roosien et al,

*Received 12 March 1998*
*Revised 14 September 1998*
*Accepted 24 November 1998*

*Correspondence to:* T Hasan

1989; Chan et al, 1991). vWF can bind directly to malignant haematopoietic cell lines at an integrin membrane receptor site (Floyd et al, 1992), facilitating interactions of tumour cells alone or tumour cell–platelet heterotypic aggregates (Jamieson, 1987) with the vascular endothelium. Adhesion to the ECM or endothelium, and subsequent migration through vessel walls, is also mediated by integrins. A role for integrin-mediated signalling in ovarian cancer has also been suggested (Buczek-Thomas et al, 1998).

In this study we report the effects of benzoporphyrin derivative monoacid (BPD-MA)-mediated PDT on integrin function and cell adhesion of ovarian cancer cells in vitro and in vivo. BPD-MA (Richter et al, 1987, 1990) was chosen because it is a photosensitizer in phase I–III clinical trials (Levy, 1994) and, unlike Photofrin, prolonged cutaneous phototoxicity with BPD-MA is not significant, and use of 690 nm light allows better penetration of tissue (Wilson, 1989).

### MATERIALS AND METHODS

### Cells and cell culture

The human ovarian cancer cell line NIH: OVCAR 3 was used in all experiments and was obtained from Dr Thomas Hamilton (Fox Chase Cancer Center, Philadelphia, PA, USA). The cells were maintained in RPMI-1640 supplemented with 10% heat-inactivated fetal calf serum (FCS; GIBCO Laboratories, Grand Island, NY) at 37°C in a 5% carbon dioxide atmosphere.

### Photosensitizer

BPD-MA was a generous gift of QLT PhotoTherapeutics Inc. (Vancouver, Canada). The compound was stored at –70°C in dimethyl sulphoxide (DMSO). It was diluted in media, and the concentration determined spectrophotometrically ($\varepsilon_{690}$ = 32 350) before each use.

### BPD-MA photosensitization

In-vitro, the cells were plated in 35-mm tissue culture dishes at least 24 h before irradiation. Densities were chosen so that the plates were approximately 50% confluent at the time of photosensitization. The cells were labelled with 8.5 μCi ml$^{-1}$ [$^{35}$S]methionine for approximately 18 h before photosensitization. Adequate incorporation of radioactivity was achieved in the growth media for this incubation period. Cells were incubated with 0.092 μmol l$^{-1}$ (0.066 μg ml$^{-1}$) BPD-MA for 3 h before irradiations. The cells were then washed with and irradiated in phosphate-buffered saline (PBS). Irradiations were 0.5 J cm$^{-2}$ (at intensities of of 0.02–0.03 W cm$^{-2}$) 690 nm light provided by an argon ion pumped dye laser (Coherent, Palo Alto, CA, USA). After irradiation, the cells were returned to media and incubated at 37°C before they were analysed for adhesion or $\beta_1$ integrin expression.

In-vivo, a nude mouse model was used (Goff et al, 1996). OVCAR 3 cells were injected intraperitoneally (i.p.) into nude mice and tumours were allowed to develop for 10–14 days. At this point, the tumour cells are largely ascitic. The animals were then injected i.p. with 2 mg kg$^{-1}$ BPD-MA 3 h before irradiations. Irradiations were done with two diffusing tip fibres and 2 ml of 0.2% intralipid as the diffusing medium as described (Molpus et al, 1996). Fluence was calculated at the skin surface at each fibre as 20 J cm$^{-2}$ using the procedure described in Lilge et al (1994). The ascitic fluid and cells were tapped 1 h after irradiation. Tumour cells were pelleted by centrifugation, treated with 0.83% $NH_4Cl$ for 5 min to lyse the red blood cell component, counted and placed in RPMI with 10% FCS and 12.5 μCi per dish [$^{35}$S]methionine at a density of 7 × 10$^5$ per 35-mm dish. Approximately 15 h later the cells were assayed for adhesion as described below.

### Adhesion assays

For adhesion assay, 96-well Falcon ‘Probind’ Immunoassay plates (Becton Dickinson, Lincoln Park, NJ, USA) were coated with ECM proteins 24 h in advance of their use. Each well was coated with 100 μl of one of the following solutions: 2 μg ml$^{-1}$ human collagen IV, human fibronectin, or human vitronectin, or 10 μg ml$^{-1}$ mouse laminin. All ECM proteins were obtained from Collaborative Biomedical Products (Becton Dickinson, Bedford, MA, USA). Dilutions were done in cold PBS. The plates were covered and stored at 4°C until use. At least 1 h before the plates were used, the ECM liquid was discarded, and the wells were blocked with 125 μl of a solution of 1% bovine serum albumin, (BSA; Sigma Chemical Co., St Louis, MO, USA) in PBS at 37°C. Immediately prior to addition of the cells to the wells, the BSA solution was poured off. The cells were harvested by trypsinization 2, 24 and 72 h after photosensitization. They were suspended in media containing 10% FCS for approximately 30 min to inactivate the trypsin. During this time live cells were counted by haemocytometer. The cells were centrifuged and resuspended in RPMI containing 1% BSA at a density of 2–5 × 10$^5$ cells ml$^{-1}$. Then, 0.10 ml of this suspension was placed in each of the wells previously prepared with ECM proteins, as well as counted in triplicate on a Beckman Model LS3801 scintillation counter to determine input radioactivity. Adhesion assay incubations were done for 2 h at 37°C before non-adherent cells were washed off with PBS. Each well was washed three times with 150 μl of PBS. The wells were examined using a microscope to ensure that this protocol removed the cells from the blank wells. A total of 150 μl of a detergent solution (0.1 mol l$^{-1}$ sodium phosphate, pH 7.5, 1 mol l$^{-1}$ sodium chloride, 10% Triton X-100, 5% sodium deoxycholate, 1% sodium dodecyl sulphate) were added to each well to lyse the cells. Then, 125 μl from each well were counted for radioactivity. In each case, the per cent adhesion was calculated as the percentage of radioactivity that bound to the matrix protein compared to the input counts. In all cases, these values were normalized to the percentage adhesion occurring with the untreated OVCAR 3 cell line. Adhesion values were typically determined by quadruplicate measurements.

### Antibody blocking

Because the $\beta_1$ integrin subunit is widely used to bind to different ECM components across a variety of cell types, use of $\beta_1$ in OVCAR 3 cells was determined by antibody blocking experiments. One hundred microlitres of 2 μg ml$^{-1}$ solutions of collagen IV and fibronectin, and 10 μg ml$^{-1}$ solution of laminin were placed in Falcon ‘Probind’ 3915 plates for 24 h at 0°C before the wells were blocked with 125 μl of 1% BSA in PBS for 1 h. At the same time, OVCAR 3 cells were allowed to incubate with 8.5 μCi ml$^{-1}$ [$^{35}$S]methionine. Afterward, the cells were harvested by trysinization after rinsing once with 10% FCS–RPMI and once with PBS without calcium and magnesium. The cells were resuspended in 10% FCS–RPMI and incubated at 37°C for 30 min. The cells were

© 1999 Cancer Research Campaign 

**Table 1** Effect of modulators of photosensitization on cellular adhesion of OVCAR 3 cells to ECM proteins[a]

| ECM protein | CAT | SOD | $NaN_3$ | $D_2O$ |
|---|---|---|---|---|
| Collagen IV | 98.76 ± 7.93 | 95.99 ± 13.47 | 125.54 ± 10.13[c] | 101.17 ± 7.40 |
| Fibronectin | 99.96 ± 9.88 | 97.75 ± 5.74 | 117.29 ± 10.82[b] | 90.93 ± 15.64 |
| Laminin | 106.26 ± 17.62 | 90.60 ± 12.04 | 158.69 ± 25.37[b] | 76.15 ± 7.41 |
| Vitronectin | 103.13 ± 3.56 | 93.89 ± 2.39 | 104.52 ± 10.40 | 90.12 ± 6.85[c] |

[a]BPD-MA photosensitization was performed in the presence and absence of the compounds listed in the table. Thirty minutes following irradiation, cells were harvested for adhesion assays. Each value represents the per cent adhesion obtained with the compound compared to that obtained when cells were photosensitized in its absence. Mean values and standard deviations from 4–5 different experiments have shown. Every experiment was performed with quadruplicate wells for each protein ($n$ = 16–20). [b]Significant at the 5% level; [c] significant at the 1% level using a paired $t$-test.

then centrifuged, and resuspended in RPMI with 1% BSA at a concentration of $5 \times 10^5$ ml$^{-1}$. Then, 2 μg ml$^{-1}$ of anti-$\beta_1$ antibody were added. Five minutes later 100 μl of cell suspension were added to the appropriate protein-coated well as in the adhesion assay. Following a similar protocol, blocking of binding to fibronectin by an antibody to the $\alpha_3$ and $\alpha_5$ subunits was also measured.

### Antibody staining for focal adhesion plaque visualization

Indirect immunofluorescence of the $\beta_1$ integrin was accomplished following the protocol of Carter et al (1990). Basically, coverslips were washed with chloroform and methanol to reduce cellular interactions with glass. The coverslips were placed in 35-mm tissue culture dishes and coated with 250 μl of 20 μg ml$^{-1}$ collagen IV at 4°C overnight. Photosensitization was performed as described above except that the fluence used was 1.0 J cm$^{-2}$ in order to obtain 50% survival. One hour after treatment the cells were collected by trypsinization, washed with RPMI containing 1% BSA and 100 μg ml$^{-1}$ soybean trypsin inhibitor (Sigma Chemical Co., St Louis, MO, USA), and 250 μl of a $1.67 \times 10^6$ cells ml$^{-1}$ dilution were placed in the coverslip dishes in RPMI with 1% BSA. The cells were allowed to incubate for 2 h before indirect immunofluorescence was done. The cells on collagen IV were washed with PBS twice, and fixed with 1% formalin for no more than 5 min. After washing with PBS with 1% filtered horse serum, the cells were stained with a 2 μg ml$^{-1}$, stock of anti-human $\beta_1$-integrin (Clone P4C10) (Telios Pharmaceuticals, Inc., San Diego, CA, USA) for 1 h at room temperature. They were then washed again, and incubated with fluorescein isothiocyanate (FITC)-conjugated conjugated goat anti-mouse IgG (Sigma Chemical Co., St Louis, MO, USA) for another hour at room temperature. The plates were washed again with PBS and the fluorescence was preserved by mounting the coverslips on slides using Gel/Mount (Biomeda Corp., Foster City, CA, USA). Fluorescence was imaged using an epi-illumination fluorescence microscope (Zeiss, Oberkochen, Germany) equipped with a Pulnix Image Intensifier, Sony video monitor, and an IBM personal computer AT. FITC was excited using a 450–490 nm bandpass filter, and emission was imaged using a 514 nm longpass filter.

### Modulation of photosensitization effects

Photosensitization experiments were performed in the presence of catalase (CAT), superoxide dismutase (SOD), sodium azide ($NaN_3$), and heavy water ($D_2O$) in order to evaluate the effects of radicals on cellular adhesion. Photosensitizations were done as described above, except that irradiations were done in the presence of 1500 U ml$^{-1}$ CAT, 500 U ml$^{-1}$ SOD, 5 mM $NaN_3$, or 90% $D_2O$. Following irradiations, the cells were placed in media and incubated at 37°C for 30 min before they were harvested for cellular adhesion analysis.

### Cellular localization of BPD-MA

#### *Cell preparation*

A total of $3 \times 10^5$ OVCAR 3 cells were plated on a microscope cover slip and incubated for at least 24 h. BPD-MA was dissolved in complete medium to a final concentration of 0.092 μmol l$^{-1}$ and incubated for 3 h. For 20 min, Rhodamine 123 (R123, Eastman Kodak, Rochester, NY, USA) was added to a final concentration of 0.01 μmol l$^{-1}$ for co-incubation (Chen, 1998). The coverslips were rinsed in PBS and mounted with PBS on a microscope slide using 0.02-mm-thick distance holders in order to prevent compression of the cells. The samples were imaged without delay.

#### *Microscopy*

A Leica confocal laser scanning microscope (Leica, Heidelberg, Germany) consisting of a Leica TDS 4D scanner attached to a Leitz DM IRD inverted microscope was operated using the TCS-NT software package. Radiation (488 nm) from an Argon laser was used for excitation. A 100 × oil immersion objective was used to image a 100 × 100 μm area at 1024 × 1024 pixels resolution. The instrument allowed simultaneous recording of three signals. One channel was used for acquiring a transmission image (differential interference contrast, DIC). The other two channels collected fluorescence signals in either the green range (530 nm bandpass filter), or the red range (590 nm longpass filter). The green and red images were combined for the overlay image.

### RESULTS

### Adhesion to ECM proteins after mild photosensitization

To investigate the effects of BPD-MA photosensitization on cellular adhesion properties of surviving tumour cells, OVCAR 3 cells were assayed after mild photosensitization. Photosensitization which yielded approximately 85% survival decreased the adhesion of OVCAR 3 cells to ECM proteins following irradiation (Figure 1). Two hours following irradiation, binding to laminin was reduced to 39% of control, binding to fibronectin was reduced by

 © 1999 Cancer Research Campaign

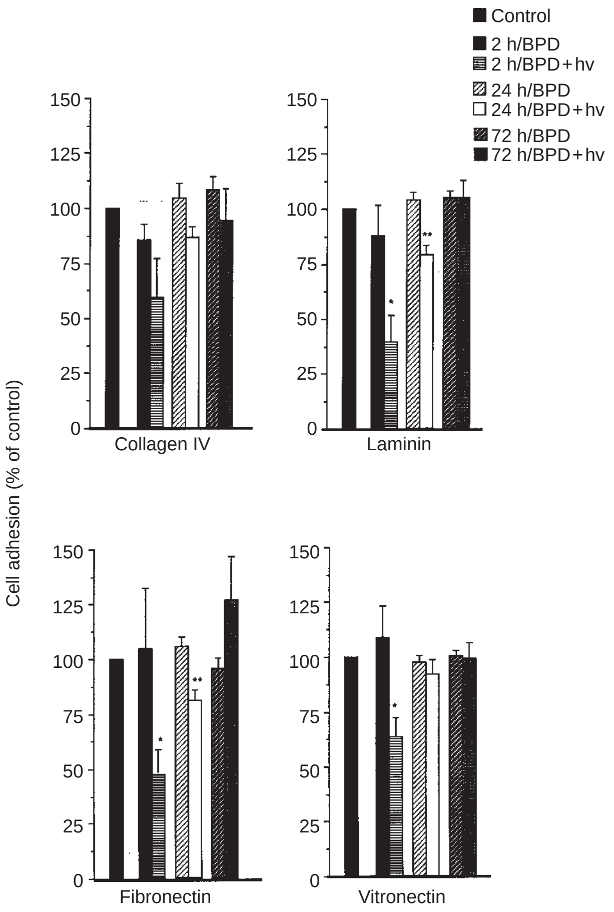

**Figure 1** Time course of photosensitization effects on cellular adhesion to ECM proteins. OVCAR 3 cells were allowed to adhere to various ECM proteins for 2 h at various times following BPD-MA photosensitization. The data obtained on cells harvested 2 and 72 h post photosensitization are compiled from four experiments, while the 24 h data are derived from five experiments. Levels of significance using a paired *t*-test: *, significant at the 5% level; **, significant at the 1% level

47% of control, while binding to collagen IV and vitronectin were least affected (59% and 64% of control respectively). By 24 h, cellular adhesion had begun to recover, such that laminin binding returned to 79% and vitronectin binding returned to 92% of the untreated control OVCAR 3 cells. By 72 h, adhesion to all of the ECM proteins returned to essentially 100% of control values. To eliminate any contribution BPD-MA dark effects might make to the data, adhesion of cells treated with BPD-MA in the absence of light was measured. These data are also presented in Figure 1. In each case, no significant effect of BPD-MA alone could be seen, indicating that the effects on cellular adhesion are the result of photosensitization and not binding of the photosensitizer to the integrin receptors directly at their sites of ECM interaction.

### Determination of integrin expression by OVCAR 3 cells

To determine which integrins the OVCAR 3 cells use to bind to ECM proteins, the cellular adhesion assay was done in the presence of competing antibody. Because the $\beta_1$ subunit is the most commonly used $\beta$ subunit, antibody against it was chosen as a major competing antibody in the adhesion and the immunofluorescence studies. These data, presented in Figure 2, show that

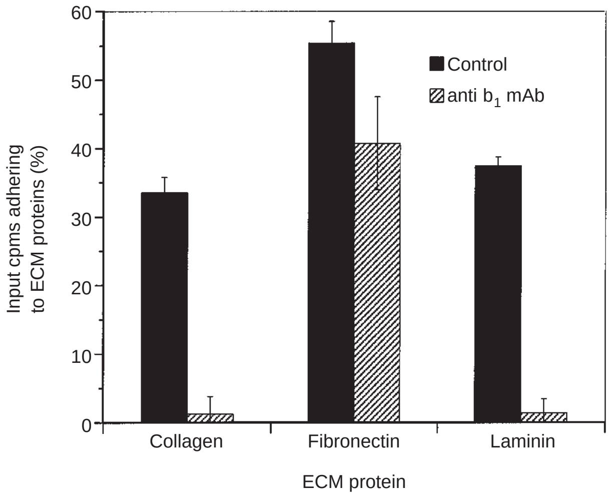

**Figure 2** Cellular adhesion in the presence of competing $\beta_1$ antibody. OVCAR 3 cells were radioactively labelled with [$^{35}$S]methionine and allowed to adhere to collagen IV, fibronectin and laminin in the presence or absence of an inhibitory anti-$\beta_1$ antibody (P4C10). Values are expressed as the percentages of radioactivity which adhered to the protein-coated wells compared to the total amount added. Values are the results of two experiments done in triplicate

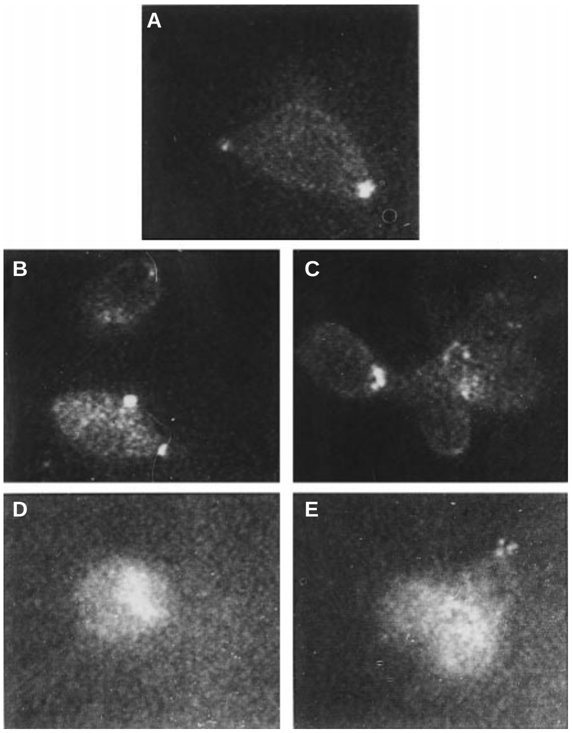

**Figure 3** Cell surface expression of $\beta_1$ integrins after photosensitization. OVCAR 3 cells were grown on glass cover slips, and treated with BPD-MA alone or with BPD-MA and light. The cells were fixed and reacted with mouse monoclonal anti-$\beta_1$ antibody (P4C10) and FITC-conjugated goat anti-mouse IgG and examined by fluorescence microscopy as described. (**A**) Untreated control cells; (**B** and **C**) treated with BPD-MA alone; (**D** and **E**) treated with BPD-MA and light

OVCAR 3 cells rely heavily on integrins possessing the $\beta_1$ subunit to bind to collagen IV and laminin; surprisingly, the $\beta_1$ antibody inhibited fibronectin binding by only about 20%.

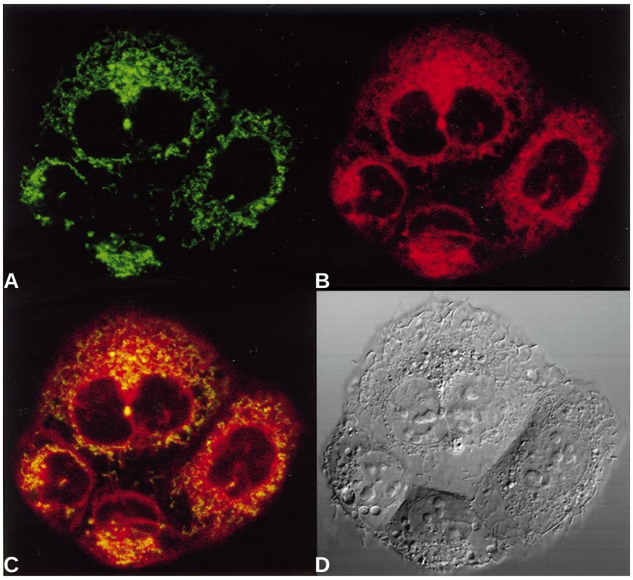

**Figure 4** Cellular localization of BPD-MA. Confocal fluorescence imaging of OVCAR 3 cells incubated for 3 h in 0.092 µmol $l^{-1}$ BPD-MA and 20 min co-incubated in 0.01 µmol $l^{-1}$ R123. (**A**) Green fluorescence of R123-stained mitochondria; (**B**) red fluorescence of BPD-MA; (**C**) combination of (**A**) and (**B**) images, where yellow indicates co-localization of red and green fluorescence; (**D**) DIC transmission image

### Effect of photosensitization on focal adhesion sites

Integrins form a link between the extracellular environment and the internal cytoplasm. In order to function in signal transduction from one environment to the other, integrins cluster to form focal adhesion sites (Kornberg and Juliano, 1991; Schwartz, 1992). Damage at these sites interferes with the ability of integrins to interact effectively with the cell's environment. To visualize damage done by photosensitization with BPD-MA, OVCAR 3 cells were stained with a mouse monoclonal anti-human $\beta_1$ integrin antibody followed by a FITC-conjugated goat anti-mouse IgG antibody, both before and 3 h following treatment (Figure 3). After treatment, $\beta_1$-containing integrins were present on the cell surface in a diffuse pattern (Figure 3 D, E): they were less organized into focal adhesion plaques (FAPs) than on untreated or cells treated with BPD-MA alone (compare Figure 3 A, B, C with D and E) These findings are consistent with a loss of integrin function resulting from photosensitization damage which manifests itself as an inability to bind to ECM proteins (Figure 1).

### Effect of quenchers and $D_2O$ on photosensitized damage to cellular adhesion

Photosensitization damage in biological systems is mediated by active molecular species, including reactive oxygen radicals. Singlet oxygen is considered to make a major contribution via a type II mechanism for a number of photosensitizers (Halliwell, 1989; Henderson and Dougherty, 1992). We have previously reported detailed analyses of the photophysical and photosensitizing properties of BPD-MA (Aveline et al, 1994, 1995) and have established the singlet oxygen yield in organic solvents to be about 0.78. One of our studies (Aveline et al, 1995) confirmed that the essentials of photophysics of this molecule remained the same upon complexation with human serum albumin. Taken together, these data would confirm an important role for singlet oxygen in the photosensitization process. However, other oxygen species, such as superoxide and hydrogen peroxide, may contribute as well (Maillard et al, 1980, Ben Hur et al, 1985; Athar et al, 1989; Kimel et al, 1989). To establish how important any of these species might be in photosensitization damage to the integrins, cellular adhesion was measured after photosensitization in the presence of various compounds which can modulate the effects of reactive oxygen species. These data are presented in Table 1.

The presence of neither catalase nor superoxide dismutase at the time of irradiation altered adhesion to ECM proteins when measured 30 min following irradiation. These data suggest that hydrogen peroxide and superoxide do not contribute substantially to the damage at the cell surface. Azide did partially reverse the effects of photosensitization on cell adhesion, implicating singlet oxygen and/or radicals as the damaging species. This reversal was significant for binding to collagen IV, fibronectin and laminin. Further studies using deuterium oxide to lengthen the lifespan of singlet oxygen potentiated effects on cellular adhesion to fibronectin, laminin and vitronectin; the effect was statistically significant only in the case of binding to vitronectin.

### Localization of BPD-MA

Co-localization using R123 demonstrated that, although BPD-MA fluorescence was very clearly localized to mitochondria, it obviously transgressed those areas co-stained by R123 (Figure 4A) and had a generally cytoplasmic pattern (Figure 4 B, C). This extra-mitochondrial staining had a diffuse and a patchy distribution (Figure 4B). Despite the non-plasma membrane localization of the photosensitizer, the adhesion and immunofluoresence data presented above indicate that at least some of the damage done with BPD-MA photosensitization is manifested at the cell surface. Since the extent of the damage was not affected by most of the modulators described in Table 1, it is possible that damage occurs at cellular sites which affect integrin function, but are sequestered at sites inaccessible to the modulators.

Prior to performing co-localization studies, we established that there was no cross-talk of the fluorescent dyes at the concentrations used (0.01 µmol $l^{-1}$, R123; 0.092 µmol $l^{-1}$, BPD-MA). This tenfold difference in concentrations reflects the more than tenfold lower fluorescence quantum yield of BPD-MA ($\varphi_F$ = 0.06) (Aveline et al, 1994) as compared to R123 ($\varphi$ = 0.99).

### In vivo adhesion characteristics of OVCAR 3 cells before and after photosensitization

As in the in vitro situation, exposure of OVCAR 3 cells to BPD-MA in vivo did not significantly affect the adhesion of the cells to the ECM proteins when compared to control ascitic cells (Figure 5). Treatment with BPD-MA and light reduced adhesion to collagen IV to 20%; fibronectin to 14%; laminin to 15%; and vitronectin to 20% of that seen with ascites control cells. More dramatic, however, was the difference in adhesion between OVCAR 3 cells in vitro and in vivo. OVCAR 3 ascites adhered to collagen IV, fibronectin, laminin and vitronectin at 20%, 7%, 11% and 18% of the respective in vitro OVCAR 3 values.

 © 1999 Cancer Research Campaign

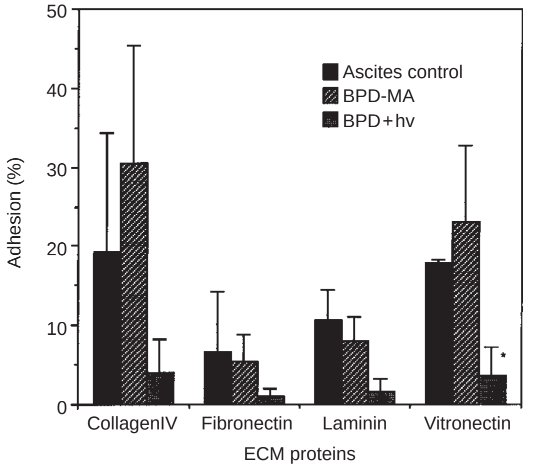

**Figure 5** Cellular adhesion to ECM proteins after in vivo photosensitization. OVCAR 3 cells were injected intraperitoneally into nude mice, and ascitic tumours allowed to develop for 14 days. The mice were then exposed to BPD-MA PDT, as described. One hour later, the ascites were harvested and the OVCAR 3 cells were radioactively labelled overnight. The following day the cells were used in adhesion assays as described above. All data are derived from triplicate samples from at least two experiments. *, significant at the 5% level using a paired *t*-test

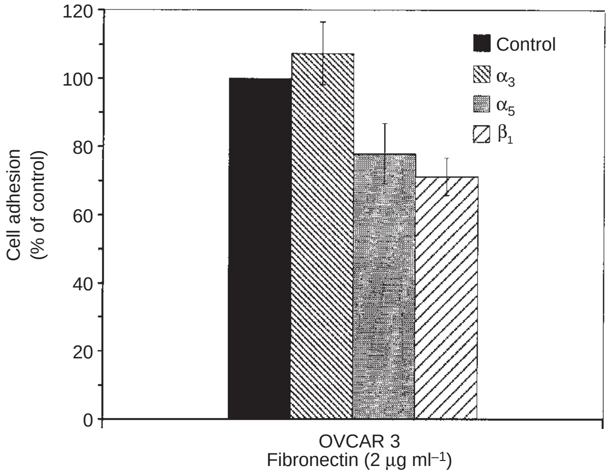

**Figure 6** Cellular adhesion in the presence of competing $\alpha_3$, $\alpha_5$ and $\beta_1$ antibodies. OVCAR 3 cells were radioactively labelled with [$^{35}$S]methionine and allowed to adhere to fibronectin in the presence or absence of an inhibitory anti-$\alpha_3$, $\alpha_5$ or $\beta_1$ antibody. Values are expressed as the percentages of radioactivity adhering to the protein-coated wells compared to the total amount added. Values are the results of two experiments done in triplicate

### DISCUSSION

The role of integrin-mediated functions, such as cellular adhesion to ECM or endothelial cells, plays an important role in many biologic processes including cell growth, differentiation, malignant transformation and metastasis. The present study is an initial investigation to determine if alterations relevant to any of these processes occur as a consequence of BPD-MA-based PDT. The findings suggest that, indeed, transient changes in integrin function and cellular adhesion do occur when OVCAR 3 cells are subject to PDT with BPD-MA.

The data presented here demonstrate that photosensitization damage to OVCAR 3 cells causes a decreased ability of the cells to bind to all four of the ECM components tested. The loss of adhesion was accompanied by the loss of FAPs and the appearance of a diffuse pattern of $\beta_1$ fluorescence after photosensitization (Figure 3). Even though FAPs disappeared, the cells retained their capacity to adhere to a collagen IV matrix (Figures 1 and 3) suggesting that either alternative β subunit integrins, or other adhesion molecules, were utilized for this binding. Consistent with this, the baseline binding of OVCAR 3 cells to fibronectin was inhibited by $\alpha_5$ and $\beta_1$ integrins (Figures 2 and 6). The implications of these data clearly need to be studied further. However, there is ample information in the literature to project on what some of these implications might be.

Numerous changes in integrin expression have been noted between normal cells and their oncogenic counterparts, although the overall picture is not straightforward, and changes in integrin expression appear to be a function of cell/tissue type. Plantefaber and Hynes (1989) reported a significant loss in expression of $\alpha_5\beta_1$ integrin (fibronectin) in transformed rodent fibroblasts (Plantefaber and Hynes, 1989). Likewise, Chinese hamster ovary cells (CHO) that have been transfected with human $\alpha_5$ and $\beta_1$ cDNAs regained their ability to synthesize $\alpha_5\beta_1$ integrin, and concomitantly lost their tumorigenicity in nude mice (Giancotti and Ruohslati, 1990). In addition, $\alpha_5\beta_1$ expression correlated with a decreased ability to migrate in vitro, a property indicative of metastatic potential in vivo. On the other hand, CHO cells with decreased fibronectin integrin receptor expression compared to the parent cell line migrated less toward fibronectin in vitro and formed faster growing tumours in vivo than the parent cells (Schreiner et al, 1989). Very few data exist on the effects of PDT on cell adhesiveness. BPD-MA PDT of fibroblast interfered with cell adhesion without altering integrin expression (Margaron et al, 1997), which is consistent with observations in our study. HPD PDT caused a reduced adhesion of colonic cancer cells to endothelial cells (Vonarx et al, 1995). Other membrane and non-membrane agents also modulate the expression of integrins (Danilov and Juliano, 1989; Conforti et al, 1990; Elices et al, 1991).

As demonstrated by comparing Figures 1 and 5, ascitic cells derived from the OVCAR 3 cell line adhere to ECM proteins far less than OVCAR 3 cells in vitro. However, once maintained in culture for a few days, ascites cells return to an in vitro adhesion pattern (data not shown). This difference probably reflects an in vivo signal which is absent in vitro, due to a change of phenotype which has been reported (Mareel et al, 1991). Alternatively, the decreased adhesion seen in vivo may result from ligand occupation of the binding sites (Wilhelm et al, 1988) and OVCAR 3 ascites are found to contain glandular structures ex vivo which are not seen when OVCAR 3 cells are maintained in vitro similar to the situation with mouse mammary cells (Aggeler et al, 1991). It appears that initiation of ovarian malignancy requires free integrin ligand sites: joint administration of ovarian tumour cells and RGD peptides prevents peritoneal seeding in experimental animals (Yamamoto et al, 1991). In the experiments presented here, the reduced capacity of the ex-vivo ascites cells to bind ECM proteins in an in vitro adhesion assay when compared to in vitro-cultured OVCAR 3 cells did not prevent tumour spread.

Occupation of the tumour cell integrin binding sites in this established malignant state may, in fact, have contributed to tumour spread, as occupation of the fibronectin integrin ligand site has been demonstrated to induce collagenase and stromelysin expression in

*© 1999 Cancer Research Campaign*

another system (Werb et al, 1989). In this model, a temporary loss in adhesive abilities after PDT in vivo would have far different effects than occupation of the ligand binding site, since the former results from a disruption of functional integrin sites and concomitent loss of the signalling mechanism, while the latter is transmitting a signal. As in the in vitro situation, however, BPD-MA PDT resulted in an overall decrease in ascitic cell binding to ECM proteins when compared to untreated controls. What the long-term consequences of this might be, can only be speculated upon. However, other studies in an ovarian cancer model and high-dose BPD-MA-based PDT (Molpus et al, 1996) did not show any increase in tumorigenicity in treated mice. The effect of low-dose PDT in this in vivo model, which would provide a better comparison with the present study, was not evaluated.

Our data (Table 1) indicate that damage done to the cellular adhesion apparatus does not occur through formation of superoxide ($O_2^-$) or $H_2O_2$ (hydrogen peroxide) species during photosensitization at the sites of the integrins, since the presence of neither SOD nor catalase affected the degree of binding to ECM proteins. While exogenous $H_2O_2$ impairs integrin function (Zhang et al, 1994), this species does not appear to damage the cell surface under BPD-MA PDT conditions. We cannot rule out that $O_2^-$ or $H_2O_2$ damage may occur internally at sites inaccessible to SOD and catalase. Furthermore, damage could be the result of conversion of $O_2^-$ or $H_2O_2$ to highly reactive oxidants such as hydroxyl radicals, $^{\bullet}OH$ (Halliwell, 1989). Data obtained in the presence of $NaN_3$ suggest the importance of singlet oxygen. These observations are consistent with our previous studies on the photophysical characterization of BPD-MA where a quantum yield of 0.78 was reported for singlet oxygen. The minimal enhancement in $D_2O$ points to the complexity of biological action of active molecular species.

In summary, this study shows that photosensitization with BPD-MA transiently reduces the binding of ovarian cancer cells to the four ECM components tested both in vitro and in vivo. Interestingly, after photosensitization, the $\beta_1$-containing integrins on the cell surface could still react with anti-$\beta_1$ antibody suggesting that the subunit was still structurally intact, although FAPs (a functional attribute) became diffuse (Figure 3). Co-localization showed that BPD-MA localizes in or around the mitochondria with no marked fluorescence of the cell membrane. Taken together, these data indicate that the loss in integrin function arises largely through intracellular damage rather than through direct damage to the integrin proteins on the cell surface. These data also imply that, if cellular adhesion to ECM and formation of FAPS are important determinants of metastasis as has been suggested (Giancotti and Ruoshlahti, 1990), then PDT clearly has the potential to modulate metastasis and this aspect of photodynamic sensitization needs further evaluation.

### ACKNOWLEDGEMENTS

The authors wish to thank QLT PhotoTherapeutics Inc. for the benzoporphyrin derivative; Coherent, Inc. for the loan of the argon dye laser; Dr Georges Wagnieres for his help with the localization and antibody experiments; Dr Christopher Lambert for useful discussions, Mr John Blake for assistance with in vivo experiments and Mrs G Vogt for transcriptional assistance. This work was supported in part by grants from the DOD-MFEL Program, Contract No. ONR N00014-94-1-0927 and the National Institutes of Health NIH 5RO1 AR40352.

### REFERENCES

Aggeler J, Ward J, Blackie M, Barcellos-Hoff MH, Strueli CH and Bissell MJ (1991) Cytodifferentiation of mouse mammary epithelial cells cultured on a reconsitututed basement reveals striking similarities to development in vivo. *J Cell Sci* **99**: 407–417

Albeda SM, Mette SA, Elder DE, Stewart R, Damjanovich L, Herlyn M and Buck CA (1990) Integrin distribution in malignant melanoma: association of the β3 subunit with tumor progression. *Cancer Res* **50**: 6757–6764

Athar M, Elmets CA, Bickers DR and Mukhtar H (1989) A novel mechanism for the generation of superoxide anions in haematoporphyrins derivative-mediated cutaneous photosensitization. Activation of the xanthine oxidase pathway. *J Clin Invest* **83**: 1137–1143

Aveline B, Hasan T and Redmond R (1994) Photophysical and photosensitizing properties of benzoporphyrin derivative monoacid ring A (BPD-MA). *Photochem Photobiol* **59**: 328–335

Aveline B, Hasan T and Redmond R (1995) The effects of aggregation, protein binding and cellular incorporation on the photophysical properties of benzoporphyrin derivative monoacid ring A (BPD-MA). *J Photochem Photobiol B: Biol* **30**: 161–169

Ben-Hur E, Carmichael A, Reisz P and Rosenthal I (1985) Photochemical generation of superoxide radical and cytotoxicity of phthalocyanines. *Int J Rad Biol* **47**: 145–147

Brem H, Gresser I, Grosfeld J and Folkman J (1993) The combination of antiangiogenic agents to inhibit primary tumor growth and metastasis. *J Ped Surg* **28**: 1253–1257

Buczek-Thomas JA, Chen N and Hasan T (1998) Integrin-mediated adhesion and signalling in ovarian cancer cells. *Cell Signal* **10**: 55–63

Carter WG, Wayner EA, Bouchard TS and Kaur P (1990) The role of integrins $\alpha_3\beta_1$ in cell-cell and cell-substrate adhesion of human epithelial cells. *J Cell Biol* **110**: 1387–1404

Chan BMC, Matsuura N, Takada Y, Zetter BR and Hamler ME (1991) In vitro and in vivo consequences of VLA-2 expression on Rhabdomyosarcoma cells. *Science* **25**: 1600–1602

Chen LB (1989) Fluorescent labeling of mitochondria. *Methods Cell Biol* **29A**: 103–123

Conforti G, Zanetti A, Pasquali-Ronchetti I, Quaglino D Jr, Neyrox P and Dejana E (1990) Modulation of vitronectin receptor binding by membrane lipid composition. *J Biol Chem* **265**: 4011–4019

Danilov YN and Juliano RL (1989) Phorbol ester modulation of integrin-mediated cell adhesion: a postreceptor event. *J Cell Biol* **108**: 1925–1933

Dedhar S and Saulnier R (1990) Alterations in integrin receptor expression on chemically transformed human cells: specific enhancement of laminin and collagen receptor complexes. *J Cell Biol* **110**: 481–489

Diprospero L, Singh G, Wilson BC and Rainbow AJ (1997) Cross-resistant to photofrin-mediated photodynamic therapy and UV light and recovery from photodynamic therapy damage in Rif-8A mouse fibrosarcoma cells measured using viral capacity. *J Photochem Photobiol B: Biol* **38**: 143–151

Dougherty TJ, Gomer CJ, Henderson BW, Jori G, Kessel D, Korbelik M, Moan J and Peng Q (1998) Photodynamic therapy [review]. *J Natl Cancer Inst* **90**: 889–905

Elices MJ, Urray LA and Hemler ME (1991) Receptor functions of the integrin VLA-3: fibronectin, collagen and laminin binding are differentially influenced by *arg–gly–asp* peptide and by divalent cations. *J Cell Biol* **112**: 169–181

Evans HH, Rerko RM, Mencl J, Clay ME, Antunez AR and Oleinick NL (1989) Cytotoxic and mutagenic effects of the photodynamic action of chloraluminum phthalocyanine and visible light in L5178Y cells. *Photochem Photobiol* **49**: 43–47

Evenson JF, Sommer S, Moan J and Christensen T (1984) Tumor-localizing and photosensitizing properties of the main components of haematoporphyrin derivative. *Cancer Res* **44**: 482–486

Fingar VH, Weiman J and Doak KW (1990) Role of thromboxane and prostacyclin release on photodynamic therapy induced tumor destruction. *Cancer Res* **50**: 2599–2603

Floyd CM, Irani K, Kind PD and Kessler CM (1992) von Willebrand factor interacts with malignant hematopoietic cell lines: evidence for the presence of specific binding sites and modification of von Willebrand factor structure and junction. *J Lab Clin Med* **119**: 467–476

Foster TH, Primavera MC, Marder VJ, Hilf R and Sporn LA (1991) Photosensitized release of von Willebrand factor from cultured human endothelial cells. *Cancer Res* **51**: 3261–3266

Foultier M-T, Vonarx-Coinsman V, Cordel S, Combre A and Patrice T (1994) Modulation of colonic cancer cell adhesiveness by haematoporphyrin derivative photodynamic therapy. *J Photochem Photobiol B: Biol* **23**: 9–17

© 1999 Cancer Research Campaign

Giancotti FG and Ruoslahti E (1990) Elevated levels of the $\alpha_5\beta_1$ fibronectin receptor suppress the transformed phenotype of Chinese hamster ovary cells. *Cell* **60**: 849–859

Glover RA, Bailey CS, Barrett KE, Wasserman SI and Gigli I (1989) Histamine release from rodent and human mast cells induced by protoporphyrin and UV light: studies of the mechanism of mast cell activation in erythropoietic protoporphyria. *Br J Dermatol* **122**: 501–512

Goff BA, Blake J, Bamberg MP and Hasan T (1996) Treatment of ovarian cancer with photodynamic therapy and immunoconjugates in a murine ovarian cancer model. *Br J Cancer* **74**: 1194–1198

Gomer CJ, Rucker N, Banerjee A and Benedict WF (1983) Comparison of mutagenicity and induction of sister chromatid exchanges in Chinese hamster cells exposed to hematoporphyrin derivative, photoradiation, ionizing radiation, or UV radiation. *Cancer Res* **43**: 2622–2627

Gomer CJ, Ferrario A and Murphree AL (1987) The effect of localized porphyrin photodynamic therapy on the induction of tumor metastasis. *Br J Cancer* **56**: 27–32

Gomer CJ, Ferrario A, Hayashi N, Rucker N, Szirth BC and Murphree AL (1988) Molecular, cellular, and tissue response following photodynamic therapy. *Lasers Surg Med* **8**: 450–463

Gomer CJ, Rucker N and Wong S (1989) Properties and applications of photodynamic therapy. *Radiat Res* **50**: 5365–5368

Gomer CJ, Luna M, Ferrario A and Rucker N (1991) Increased transcription and translation of heme oxygenase in Chinese hamster fibroblasts following photodynamic stress or Photofrin II incubation. *Photochem Photobiol* **53**: 275–279

Halliwell B (1989) How to characterize a biological antioxidant. *Free Rad Res Comms* **9**: 1–32

Hasan T and Parrish JA (1996) Photodynamic therapy of cancer. In: *Cancer Medicine*, 4th edn, Holland JF, Frei E III, Bast RC Jr, Kufe DW, Morton DL and Weichselbaum RR (eds), pp. 739–751. Baltimore: Williams and Wilkins

Henderson BW and Donovan JM (1989) Release of prostaglandin $E_2$ from cells by photodynamic treatment in vitro. *Cancer Res* **49**: 6896–6900

Henderson BW and Dougherty TJ (1992) How does photodynamic therapy work? *Photochem Photobiol* **55**: 145–157

Ingber D and Folkman J (1988) Inhibition of angiogenesis through modulation of collagen metabolism. *Lab Invest* **59**: 44–51

Jamieson GA (1987) Mechanisms of interaction between platelets and tumor cells using homologous human systems. In: *Hemostasis and Cancer*, Musbek L (ed.) Boca Raton, FL: CRC Press

Karpatkin S, Pearlstein E, Abvrogio C and Coller BS (1988) Role of adhesive proteins in platelet tumor interaction in vitro and metastasis formation in vivo. *J Clin Invest* **81**: 1012–1019

Kimel S, Tromberg BJ, Roberts WG and Berns MW (1989) *Photochem Photobiol* **50**: 175–183

Kornberg L and Juliano RL (1991) Signal transduction from the extracellar matrix: the integrin-tyrosine kinase connection. *Trends Pharmacol* **13**: 95

Levy JG (1994) Photosensitizers in photodynamic therapy. *Sem Onc* **21**: 5–10, Suppl. 15

Lilge L, Dabrowski W, Holdsworth DW, Blake J, Kato D, Wilson BC and Hasan T (1994) Light delivery and dosimetry for photodynamic therapy in an ovarian cancer mouse model. *Proc SPIE* **2133**: 150–161

Maillard P, Krausz P, Gianotti C and Gaspard S (1980) Photoinduced activation of molecular-oxygen by various porphyrins, phthalocyanines, pyridinoporphyrazins and their metal derivatives. *J Orgmet* **197**: 285–290

Malik Z and Djaldetti M (1980) Destruction of erythroleukemia, myelocytic leukemia and Burkitt lymphoma cells by photoactivated porphyrin. *Int J Cancer* **26**: 495–500

Mareel MM, Behrens J, Birchmeier W, Bruyne GK, Vlemickx L, Hoogewijis A, Fiers WC and Van Roy FM (1991) Down-regulation of E-cadherin expression in Madin Darby canine kidney (MDCK) cells inside tumors of nude mice. *Int J Cancer* **47**: 922–928

Margaron P, Sorrent R and Levy JG (1997) Photodynamic therapy inhibits cell adhesion without altering integrin expression. *Biochim Biophys Acta* **1359**: 200–210

Moan J and Vistnes AI (1986) Porphyrin photosensitization of proteins in cell membranes as studied by spin-labeling and by quantification of DTNB-reactive SH-groups. *Photochem Photobiol* **44**: 15–19

Molpus KL, Kato D, Lilge L, Hamblin MR, Bamberg M and Hasan T (1996) Intraperitoneal photodynamic therapy of human epithelial ovarian carcinomatosis in a xenograft murine model. *Cancer Res* **56**: 1075–1082

Plantefaber LC and Hynes RO (1989) Changes in integrin receptors on oncogenically transformed cells. *Cell* **56**: 281–290

Reich R, Royce L and Martin G (1989) Eicosapentaenoic acid reduces the invasive and metastatic activities of malignant tumor cells. *Biochem Biophys Res Commun* **160**: 559–564

Richter AM, Kelly B, Chow J, Liu DJ, Towers GHN, Dolphin D and Levy JG (1987) Preliminary studies on a more effective phototoxic agent than hematoprophyrin. *J Natl Cancer Inst* **79**: 1327–1332

Richter AM, Cerruto-Sola S, Sternberg ED, Dolphin D and Levy JG (1990) Biodistribution of tritiated benzoporphyrin derivative ($^3$H-BPD-MA), a new potent photosensitizer in normal and tumor bearing mice. *J Photochem Photobiol B: Biol* **5**: 231–244

Roosien FF, De Ruk D, Bikker A and E Ross (1989) Involvement of LFA-1 in lymphoma invasion and metastasis demonstrated with LFA-1 deficient mutants. *J Cell Biol* **108**: 1979–1985

Schreiner C, Fisher M, Hussein S and Juliano RL (1989) Increased tumorigenicity of fibronectin receptor deficient Chinese hamster ovary cell variant. *Cancer Res* **51**: 1738–1740

Schreiner CL, Bauer JS, Danilov YN, Hussein S, Sczekan MM and Juliano RL (1991) Isolation and characterization of Chinese hamster ovary cell variants deficient in the expression of fibronectin receptor. *J Cell Biol* **109**: 3157–3167

Schwartz MA (1992) Transmembrane signalling by integrins. *Trends Cell Biol* **2**: 304–308

Singh G, Wilson BC, Sharkey SM, Browman GP and Deschamps P (1991) Resistance to photodynamic therapy in radiation induced fibrosarcoma-1 and Chinese hamster ovary multi-drug-resistant cells in vitro. *Photochem Photobiol* **54**: 307–312

Vonarx V, Foultier MT, Xavier De Brito L, Anasagasti L, Morlet L and Patrice T (1995) Photodynamic therapy decreases cancer colonic cell adhesiveness and metastatic potential. *Res Exp Med* **195**: 101–116

Werb Z, Tremble PM, Behrendtsen O, Crowley E and Damsky CH (1989) Signal transduction through the fibronectin receptor induces collagenase and stromelysin gene expression. *J Cell Biol* **109**: 877–889

Wilhelm O, Hafter R, Coppenrath E, Pflanz MA, Schmitt M, Babic R, Linke R, Gossner W and Graeff H (1988) Fibrin-fibronectin compounds in human ovarian tumor ascites and their possible relation to tumor stroma. *Cancer Res* **48**: 3507–3514

Wilson BC (1989) Photodynamic therapy: light delivery and dosage for second-generation photosensitizers. In: *Photosensitizing Compounds: Their Chemistry, Biology and Clinical Use*. Wiley, Chichester (Ciba Foundation Symposium 146), p 60–77

Yamamoto K, Murae M and Yasuda M (1991) RGD-containing peptides inhibit experimental peritoneal seeding of human ovarian cancer cells. *Acta Obstetrica et Gynacologica Japonica* **43**: 1687–1692

Young MR, Young ME and Wepsic HT (1987) Effect of prostaglandon $E_2$-producing nonmetastatic Lewis lung carcinoma cells on the migration of prostaglandin $E_2$-responsive metastic Lewis lung carcinoma cells. *Cancer Res* **47**: 3679–3683

Zhang Z, Turner DC, Drzewiecki GJ, Hinshaw DB and Hyslop PA (1994) Impairment of integrin-mediated cell-matrix adhesion in oxidant-stressed PC12 cells. *Brain Res* **662(1–2)**: 189–197

*© 1999 Cancer Research Campaign*
